# The Role of MicroRNA in the Pathophysiology and Diagnosis of Viral Myocarditis

**DOI:** 10.3390/ijms252010933

**Published:** 2024-10-11

**Authors:** Ewelina Młynarska, Krzysztof Badura, Szymon Kurciński, Julia Sinkowska, Paulina Jakubowska, Jacek Rysz, Beata Franczyk

**Affiliations:** 1 Department of Nephrocardiology, Medical University of Lodz, Ul. Zeromskiego 113, 90-549 Lodz, Poland; 2 Department of Nephrology, Hypertension and Family Medicine, Medical University of Lodz, Ul. Zeromskiego 113, 90-549 Lodz, Poland

**Keywords:** viral myocarditis, microRNA, myocarditis pathophysiology, myocarditis diagnosis, non-coding RNA

## Abstract

Myocarditis is a non-ischemic condition with a heterogeneous etiology, clinical course and prognosis. The most common etiology of myocarditis are viral infections, whereas the most severe complications are acute and chronic heart failure and sudden cardiac death. The heterogeneous clinical course of the disease, as well as the availability and costs of diagnostic tools such as cardiac magnetic resonance and endomyocardial biopsy, hinder the diagnosis of myocarditis and its underlying cause. Non-coding RNAs such as micro-RNAs (miRNAs; miR) have been shown to be involved in the disease’s pathophysiology; however, their potential in disease diagnosis and treatment should also be considered. Non-coding RNAs are RNAs that are not translated into proteins, and they have the ability to regulate several intracellular pathways. MiRNAs regulate gene expression by binding with their targets and inhibiting protein synthesis by interfering with the translation of coding genes or causing the degradation of messenger RNA. Several miRNAs, such as miR-1, -133, -21, -15, -98, -126, -155, -148, -203, -208, -221, -222, -203 and -590, have been shown to be involved in the pathophysiology of viral myocarditis (VMC), and some of them have been shown to have diagnostic abilities. This article summarizes the available data on miRNAs and their associations with VMC.

## 1. Introduction

Myocarditis can be defined as an inflammatory disease of the heart secondary to a wide range of underlying causes, such as infections, autoimmune disorders, toxic injuries and radiation [1,2,3]. It can impair the mechanical and bioelectric function of the heart, which may lead to heart failure, arrhythmias and/or conduction disorders. This condition may be acute and may self-resolve. The severe and life-threatening form of acute heart injury is fulminant myocarditis, which is characterized by a rapid progression of myocardial inflammation and can lead to a significant impairment of cardiac function. Fulminant myocarditis is associated with hemodynamic instability, which leads to cardiogenic shock [4]. The main long-term complication of myocarditis is dilated cardiomyopathy presenting as chronic heart failure [5]. The clinical course of myocarditis remains highly variable, as patients present with symptoms like fatigue, shortness of breath, fever, chest pain, palpations, loss of consciousness or flu-like symptoms like headache, body ache and joint pain.

**Figure 1:**
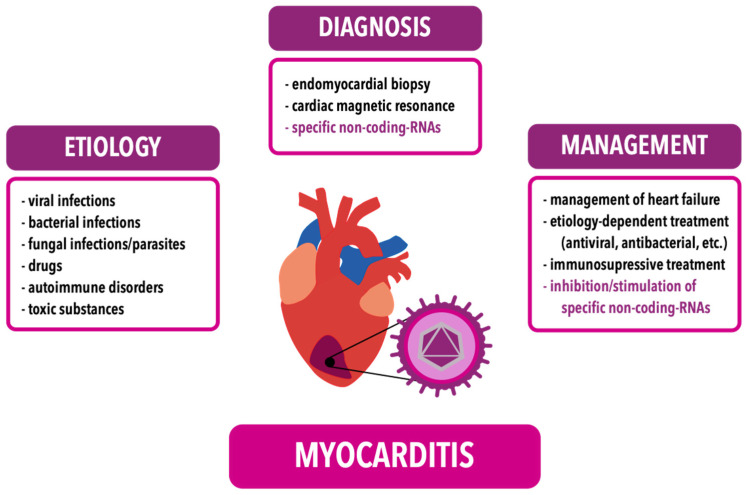
Etiology, diagnosis and management of myocarditis and potential fields of use of non-coding-RNAs. This article focuses on specific non-coding-RNAs in VMC, as shown in the figure (purple text).

According to recent data, viral infections are thought to be the most common cause of myocarditis in the adult and pediatric populations; however, infective myocarditis can also be caused by bacteria, fungi or parasites [3,6,7]. VMC has an incidence rate of 10 to 22 per 100,000 individuals [6]. Endomyocardial biopsy is the gold standard for myocarditis diagnosis; however, unfortunately, it has a limited clinical application, so the real incidence rate is underestimated. Currently, cardiac magnetic resonance (CMR) seems to be more applicable, due to its non-invasive properties and higher availability, unlike biopsy. As of now, there is no specific blood test to diagnose VMC [4]. Therefore, novel methods for VMC diagnosis should be evaluated. Recently published studies suggested the potential diagnostic abilities of non-coding RNAs (ncRNAs), which can be sampled from peripheral blood or tissues [5]. General information about the etiology, diagnosis and management of myocarditis is compendiously presented in Figure 1. The aim of this article is to summarize the currently available knowledge on miRNAs in VMC pathophysiology and emphasize the potential role of specific miRNAs as a diagnostic tool in myocarditis. Moreover, understanding the interactions between miRNAs and pathophysiological pathways may be crucial for the development of novel therapies applicable to VMC.

## 2. Non-Coding RNAs—An Overview

NcRNAs are types of RNAs that are not translated into proteins; they include ribosomal RNA (rRNA), transfer RNA (tRNA), small-nuclear RNA (snRNA), circular RNA (circRNA), piwi-interacting RNA (piRNA), microRNA (miRNA; miR) and long non-coding RNA (lncRNA) [8]. Some ncRNAs change notably during disease and have good stability in peripheral circulation, which gives them a great potential role in VMC diagnosis and treatment. In the following section, only miRNA has been described, as the role of other ncRNAs in VMC remains out of the scope of this article.

### MicroRNA

MiRNAs are small, non-coding ribonucleic acids ranging from 20 to 25 nucleotides in length. They are functionally involved in the regulation of gene expression through a post-transcriptional mechanism [9]. MiRNAs bind with their completely or partially complementary mRNA targets, which leads to inhibition of translation of mRNA and/or degradation of mRNA. This can cause a suppression of protein production.

In humans, the production of mature miRNAs starts in the nucleus with the transcription of miRNA genes by RNA polymerase II (Pol II). MiRNA genes are transcribed into molecules called pri-miRNAs. These transcripts have a cap structure at the 5′ end and a poly(A) tail at the 3′ end, both of which are specific targets for Pol II. Then, pri-miRNAs are subjected to RNAse III endonuclease, called the DROSHA enzyme, and the precursor miRNA (pre-miRNA) is created. Then, it is released into the cytoplasm. Further processes on pre-miRNA take place in the cytoplasm with the participation of cytoplasmic RNAses. At the end of these processes, mature and functional miRNAs emerge. These mature miRNAs are incorporated into the RISC (RNA-induced silencing complex). This incorporation leads the RISC to the target mRNA [10,11,12,13,14].

**Figure 2:**
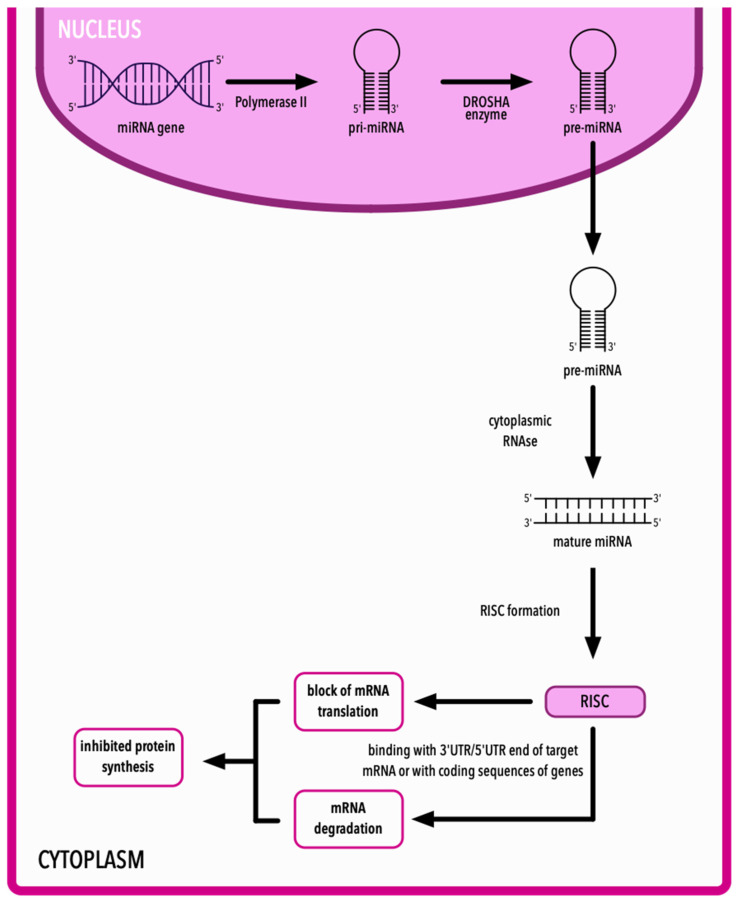
Mature miRNA synthesis, RISC formation and its impact on mRNA and protein synthesis. mRNA—messenger RNA; miRNA—microRNA; RISC—RNA-induced silencing complex; UTR—untranslated region.

Most miRNAs interact with the 3′ untranslated regions (UTRs) of target mRNA. Apart from the 3′UTR end, miRNAs can also bind with the 5′UTR end of their target mRNA and with the coding sequences of genes [15,16]. By binding with their targets, miRNAs inhibit protein synthesis by interfering in the translation of coding genes or by causing the degradation of mRNA. General mechanisms explaining mechanism of action of miRNAs are shown on Figure 2.

## 3. MicroRNA and the Pathophysiology of Viral Myocarditis

**Figure 3:**
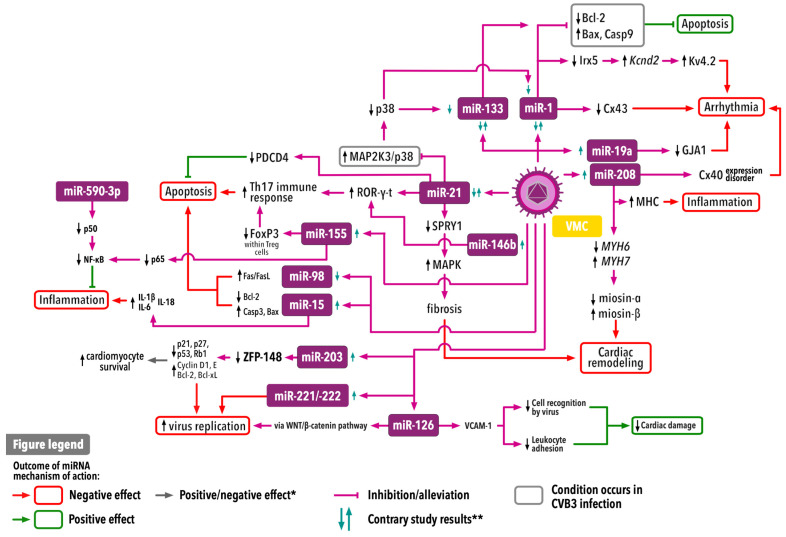
Simplified mechanisms of action of specific miRNAs and their influence on VMC. * Outcome may be positive or negative depending on the case. ** Currently available data do not allow us to conclude whether a specific miRNA is upregulated or downregulated in VMC (see Section 3). Cx—connexin; CVB3—Coxsackie B3 virus; GJA1—gap junction protein α1; IL—interleukin; NLRP3—NLRP3 inflammasome; MHC—major histocompatibility complex; miR—microRNA; PDCD4—programmed cell death protein 4; ROR—RAR-related orphan receptor; SPRY1—Sprouty RTK signaling antagonist 1; VCAM-1—vascular cell adhesion molecule 1; VMC—viral myocarditis; ZFP-148—zinc finger protein 148.

Most studies assessing the role of miRNA in myocarditis are based on animal models with Coxsackie B3 (CVB3)-induced myocarditis. CVB3 is a cardiotropic virus belonging to the Coxsackie B virus group of the Enterovirus genus within the Picornaviridae family. CVB3 frequently causes myocarditis in humans; therefore, it is commonly used to create murine models of VMC, as CVB3 may induce VMC in susceptible strains of mice. Moreover, the pathophysiological and pathomorphological parameters of these models correspond with those observed in CVB3-induced VMC in humans [17]. Although the mechanisms described in these studies may apply to myocarditis caused by other viruses, the exact pathophysiological mechanisms that occur in non-CVB3-associated myocarditis remain unexplored. The general mechanisms of action of specific miRNAs in VMC are presented in Figure 3.

### 3.1. miR-1 and miR-133

Recent evidence suggests a significant role of miR-1 and miR-133 in physiological as well as pathological conditions involving the myocardium. It has been shown that in physiological conditions, muscle-specific miRNAs such as miR-1 and miR-133 are key regulating factors that are involved in the proliferation and apoptosis of myocardial cells during cardiac development [18,19]. Moreover, several studies have revealed associations between cardiac diseases such as heart failure, myocardial hypertrophy and cardiac arrhythmias and differences in the expression of miR-1 and miR-133 [20,21,22].

An increased expression of miR-1 has been shown in models of VMC, whereas an increase in miR-1 was associated with decreased connexin 43 (Cx43) expression [23,24]. Cx43 is a gap junction-forming protein within atrial and ventricular cardiomyocytes that allows for cell-to-cell cytoplasmatic communication between cardiomyocytes. The overexpression, as well as underexpression, of Cx43 is associated with impaired impulse generation and conduction due to the heterogenous expression of multiple connexins. This phenomenon may constitute an underlying cause of atrial and ventricular arrhythmias that may lead to sudden cardiac death (SCD) [25,26,27].

On the other hand, a study by Li et al. [28] has shown a downregulation of both miR-1 and miR-133, whereas Besler et al. [29] have shown miR-133a to be increased in VMC cardiomyocytes. MiR-1 and miR-133 attenuate apoptosis and cardiac dysfunction through the regulation of pro- and anti-apoptotic-related gene expression. In VMC, the expression of pro-apoptotic genes such as *Bax* and *Casp9* tends to be increased, whereas the expression of the anti-apoptotic *Bcl-2* gene remains decreased. MiR-1 and miR-133 prevent changes in pro- and anti-apoptotic gene expression in VMC, which may suggest a significant role of miR-1 in myocardial cell apoptosis associated with VMC. The important role of miR-1 and miR-133 in cell cycle regulation has been shown by Zhang et al. [30]. MiR-1 and miR-133 may directly and indirectly target Cyclin D1, which facilitates the progression from the G1 phase to the S phase of the cell cycle [30]. Thus, the downregulation of these two miRNAs may lead to an enhanced proliferation of cardiomyocytes, which may cause cardiac remodeling, which constitutes a complication of myocarditis. More specific mechanisms of this phenomenon comprise the ability of miR-133 to target Sp1, a factor mediating Cyclin D1 transcription, whereas Cyclin D1 constitutes a target for miR-1 [30]. Moreover, there are several links between miR-1 and potassium and calcium channel function. In VMC, an aberrant expression of *Kcnj2* and *Kcnd2* genes occurs. *Kcnj2* encodes Kir2.1, whereas *Kcnd2* encodes Kv4.2, both of which are components involved in the repolarization of potassium channels. The results of this study also indicate a negative correlation between the levels of miR-1 and Irx5, which is a transcriptional repressor of the *Kcnd2* gene [28].

Apart from VMC, the role of miR-1, miR-133a-2 and miR-133b in chronic Chagas disease cardiomyopathy was analyzed by Ferreira et al. [31]. The findings of their study support the finding that, in an inflamed myocardium caused by *Trypanosoma Cruzi* infection, several miRNAs, such as miR-1, miR-133a-2 and miR-133b, tend to be overexpressed [31].

Currently available data on miR-1 and miR-133 are limited, and contrary results have been published. Based on the understanding that miR-1 remains increased in VMC, the risk of cardiac arrhythmias may be increased due to Cx43 downregulation; however, in this case, the apoptosis of cardiomyocytes should be alleviated, suggesting a potentially protective role of both miR-1 and miR-133. On the other hand, a decreased expression of miR-1 and miR-133 may be associated with enhanced apoptosis and more excessive cardiac damage. Further studies are required to establish the time-dependent changes in both miR-1 and miR-133 expression in VMC.

### 3.2. miR-21

A study by Liu et al. [32] showed an increased expression of miR-21 in VMC, and the significance of this phenomenon was confirmed with miR-21 inhibitors, which attenuated the severity of myocarditis. The underlying mechanism of this phenomenon involves the ability of miR-21 to indirectly regulate Th17 lymphocyte function, which plays an important role in the development of VMC [33]. Th17 lymphocytes are CD4+ Th cells with the ability to produce IL-17, which is involved in VMC [33]. The authors showed that interleukin-17 (IL-17) and RAR-related orphan receptor (ROR)-γ-t expression positively correlated with miR-21 expression, whereas miR-21 inhibitors may have decreased the expression of ROR-γ-t [32]. ROR-γ and ROR-γ-t are both encoded by the *RORC* gene and constitute transcription factors responsible for Th17 lymphocyte differentiation. Moreover, an increased expression of ROR-γ-t leads to IL-17 overexpression [34].

Interestingly, He et al. [35] obtained the opposite result, suggesting a potential protective role of miR-21 in CVB3-induced myocarditis. The authors found that miR-21 expression was significantly decreased in myocarditis; however, the expression of miR-21 varied in terms of its severity. The lowest expression of miR-21 was observed in severe myocarditis cases, whereas in mild myocarditis, its expression was only slightly decreased. Moreover, treatment with miR-21 alleviated myocarditis. It was suggested that the underlying mechanism of this phenomenon is based on miR-21’s ability to target programmed cell death 4 (PDCD4) protein, which is a pro-apoptotic factor. The inhibition of PDCD4 prevents cardiomyocytes from undergoing apoptosis [35].

The results of both studies presented above should be interpreted carefully, as several differences in methodology and limitations can be found between them. It should be noted that their opposite findings may be caused by the different times after infection at which the hearts of infected mice were collected. In the study conducted by He et al. [35], miR-21 expression was analyzed 7 days after infection, whereas in the study of Liu et al. [32], surviving animals were sacrificed on day 17. It has been reported that mice strains such as A/J (H-2^a^) and BALB/c (H-2^d^) develop acute myocarditis in 10 up to 17 days after infection [36]. To date, there have been no studies that assess changes in the expression of miR-21 over time; however, it is possible that the expression of miR-21 is different on days 7 and 17 after infection. Further studies are required to determine changes in miR-21 expression over time. The presented studies are compared in Table 1.

Another study that aimed to assess the role of miR-21 in VMC showed that Sprouty RTK signaling antagonist 1 (SPRY1) is a target for miR-21 and that miR-21 is a post-translational inhibitor of sprouty protein (SPRY), whereas the inhibition of SPRY protein results in MAPK signaling pathway enhancement [37]. SPRY is a negative feedback regulator of MAPK with a mechanism of action based on inhibition through the CKD/MAPK/GSK3/CLK kinase group. Through this pathway, SPRY’s signal can be transduced to the nucleus to regulate cell growth, transformation, differentiation, proliferation and apoptosis [37]. Moreover, it should be noted that SPRY regulates the expression of the collagen gene. Inhibition of SPRY1 caused by miR-21 leads to MAPK enhancement, which contributes to myocardial fibrosis and remodeling, which are associated with the progression of VMC to dilated cardiomyopathy [37]. On the other hand, a study assessing the associations between miRNA and the characteristics of patients with inflammatory and dilated cardiomyopathy did not show a significant difference in miR-21 between patients with and without myocardial fibrosis [38].

Another study assessing the potential protective role of miR-21 in CVB3-induced myocarditis revealed that miR-21 targets the MAP2K3/p38 MAPK signaling pathway. MAP2K3 is upregulated in CVB3 infection and seems to be crucial for CVB3 infection. CVB3 has an ability to activate the MAPK p38 subfamily, which is involved in viral replication, the inflammatory response and cell apoptosis [39]. Moreover, MAP2K3 activation is crucial to maintaining p38 activation [39,40]. Thus, the authors suggested that miR-21 may have a protective role in VMC by decreasing the activity of the MAP2K3/p38 MAPK signaling pathway, which subsequently prevents cardiomyocytes from undergoing apoptosis [39]. Interestingly, p38 activation seems to be crucial for miR-1 and miR-133a transcription [30]. Thus, a potential indirect interaction between miR-21 and miR-1/133a may occur, where miR-21-mediated inhibition of p38 may decrease miR-1/133a transcription. Further studies are required to determine the interaction between these two miRNAs in VMC.

Recent data indicate a potential interaction between miR-21 and lncRNA. LncRNAs may act as “miRNA sponges” by reversing miRNAs’ effects. The results of a recently published study by He et al. [41] suggest that lncRNA-MEG3, which is upregulated in CVB3-induced myocarditis, may have an ability to sponge miR-21. LncRNA-MEG3 transcript variant 1 directly modulates the expression of miR-21, which may lead to increased activity of the MAPK p38 subfamily, which contributes to cardiomyocyte necrosis, apoptosis and an enhanced inflammatory response [41].

Several theories have been proposed to explain the role of miR-21 in VMC. Its potentially protective role in CVB3-induced myocarditis involves the ability of miR-21 to target pro-apoptotic PDCD4 and the MAP2K/p38 pathway, preventing apoptosis. On the other hand, an increased expression of miR-21 may lead to VMC complications such as myocardial remodeling by targeting SPRY, which subsequently leads to increased myocardial fibrosis. The harmful effect of miR-21 may be explained by its ability to increase the expression of ROR-γ-t, leading to enhanced inflammation. Further studies are required to determine the influence of myocarditis severity on miR-21 expression and changes in expression over time.

### 3.3. miR-15

**Figure 4:**
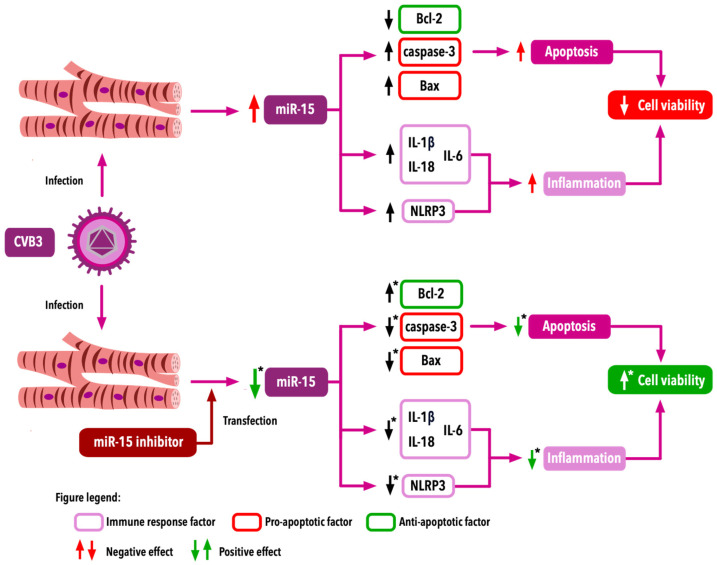
Mechanisms leading to cardiomyocyte viability changes in CVB3-induced myocarditis. * in comparison to CVB3-infected group and negative control group (CVB3-infected cells transfected with miR-15 inhibitor; negative control). CVB3—Coxsackie B3 virus; IL—interleukin; NLRP3—NLRP3 inflammasome; miR-15—microRNA-15.

A study by Tong et al. [42] revealed an increased expression of miR-15 in a model of CVB3-induced VMC. The findings of their study suggest a significant role of miR-15 in VMC, as the apoptosis of CVB3-infected cells was associated with increased miR-15 expression, whereas miR-15 inhibitors increased the viability of infected cells. Moreover, the inhibition of miR-15 was associated with a reduction in CVB3-induced apoptosis. This phenomenon can be explained by the reduction in CVB3-associated Bcl-2 decrease and suppressed increase in caspase-3 and Bax after miR-15 inhibition. According to the inflammatory response, a significant reduction in increased levels of IL-1β, IL-6 and IL-18 can be observed after miR-15 inhibition. The inflammatory response in CVB3-induced myocarditis is based on the activation of the NLRP3 inflammasome. The results of the study indicate that after CVB3 infection, levels of NLRP3, caspase-1 and p20 are increased, whereas the increase in these factors is reduced due to miR-15 inhibition. The potential mechanism of NLRP3 inhibition comprises targeting the interaction between miR-15 and nod-like receptor X1 (NLRX1), which is a negative regulator of inflammation [42]. The mechanisms leading to decreased cell viability and the potential role of miR-15 are presented in Figure 4.

### 3.4. miR-98

To the best of the authors’ knowledge, only two studies have assessed the potential role of miR-98 in myocarditis [43,44]. The study by Zhang et al. [43] has shown that miR-98 expression was severely decreased in patients with myocarditis. Moreover, the results of their study indicate that *FAS*/*FASL* genes constitute a target for miR-98 [43]. Fas/FasL molecules, which constitute a product of the expression of *FAS*/*FASL* genes, constitute costimulatory molecules involved in the induction of cell apoptosis [45]. Interestingly, it has been suggested that the Fas/FasL pathway may play a critical role in massive myocardial necrosis development in the course of VMC. The ability of miR-98 to block this pathway indicates a potentially protective effect of miR-98, as it may prevent fulminant cardiac damage [46].

The second study assessed the role of miR-98 in autoimmune myocarditis [44]. The authors of the study hypothesized that miR-98 may reduce the expression of IL-10 in IL-10-producing B lymphocytes, also called B10 cells. Interestingly, the study showed a decreased frequency of B10 cells in the inflamed myocardium, which was accompanied by increased miR-98 within B10 cells [44]. Moreover, an increase in serum TNF-α was observed and linked with an upregulated expression of miR-98 among naïve B lymphocytes [44]. Based on these findings, Chen et al. [44] proposed the theory that increased TNF-α causes an increase in miR-98 in B cells, whereas miR-98 suppresses IL-10 formation, which subsequently leads to decreased immune tolerance and enhanced inflammation.

According to the presented theories, miR-98 may have a protective role in VMC by decreasing the expression of Fas/FasL, thus leading to apoptosis and necrosis prevention, especially in severe, fulminant myocarditis. On the other hand, miR-98 in autoimmune myocarditis may be involved in decreased immune tolerance via targeting IL-10 within B lymphocytes.

### 3.5. miR-126

MiR-126 has been found to be upregulated in CVB3-induced myocarditis [47]. The induction of miR-126 was associated with the activation of transcription factor E-twenty-six (ETS) via ERK1/2 signaling. Moreover, miR-126 may induce CVB3 replication and cell death through ERK1/2 and WNT/β-catenin crosstalk. The underlying mechanism of this phenomenon is based on the ability of miR-126 to target SPRED1 protein, low-density lipoprotein receptor-related protein 6 (LRP6) and Wnt responsive Cdc42 homolog 1 (WRCH) [47].

Newer studies confirm increased levels of miR-126 within myocardial cells in inflamed cardiomyopathy [29,38]. It should be noted that plasma levels of miR-126 were higher in patients with inflamed cardiomyopathy without signs of fibrosis when compared to a group with diagnosed myocardial fibrosis; however, no correlation between miR-126 and C-reactive protein has been observed [29,38].

Apart from the role of miR-126 in immune response regulation in CVB3-induced myocarditis, miR-126 remains the most widely studied miRNA in vascular biology [48]. It has been shown that several myocarditis-related viruses, such as Cytomegalovirus (CMV), Coxsackievirus, Parvovirus B19 (PVB19) and Herpes simplex virus, have an ability to infect endothelial cells of the cardiac microvasculature [49]. The infection and subsequent damage of cardiac cells (especially the vascular endothelium and cardiomyocytes) leads to an increased expression of chemokines and cytokines, subsequently leading to an increased expression of vascular cell adhesion molecule 1 (VCAM-1), as well as other adhesion molecules, such as intercellular adhesion molecule 1 (ICAM-1) and E-selectin, in the cardiac vascular endothelium [49]. It has been shown that miR-126 inhibits the expression of VCAM-1, which mediates leukocyte adhesion to the endothelium [50]. Pathogens such as CVB3, rhinovirus and adenovirus utilize ICAM-1 and VCAM-1 to recognize and infect endothelial cells [49]. Thus, it is possible that miR-126 may alleviate cardiac damage in VMC via targeting VCAM-1, subsequently leading to decreased leukocyte-mediated damage. Moreover, miR-126-mediated downregulation of VCAM-1 may cease positive feedback, which exacerbates infection by allowing viruses to recognize and enter cardiac cells via adhesion molecules.

Recent data suggest a protective, as well as potentially harmful, role of miR-126 in VMC. The ability of miR-126 to induce virus replication and cell death via ERK1/2 and WNT/β-catenin crosstalk may exacerbate myocarditis due to an excessive loss of cardiomyocytes and increased virus replication. According to vascular mechanisms, an increased expression of miR-126 may be associated with the alleviation of inflammation and decreased cell recognition and infection by the virus as it targets VCAM-1.

### 3.6. miR-155 and miR-148

The expression of both miR-155 and miR-148a was found to be increased in VMC [14,38,51,52]. Several studies indicate that miR-155 is involved in VMC pathogenesis, especially in the modulation of the immune reaction associated with myocardial dysfunction [14,51,52]. MiR-155 is associated with an adverse immune reaction in VMC as it regulates T lymphocyte activation. Silencing of miR-155 in VMC has been associated with a decreased activation of splenic CD4+ and CD8+ T cells, decreased proliferation of splenic CD4+ T cells and decreased production of proinflammatory IFN-γ, whereas the production of anti-inflammatory IL-4 and IL-13 has been found to be increased. Moreover, miR-155 may also be involved in the process of macrophage polarization in the CVB3-infected myocardium. Silencing of miR-155 was associated with M2 macrophage polarization, consistent with increased IL-4 and IL-13 levels, which are responsible for M2 polarization, wherein an increased phosphorylation of STAT6, a transcription factor for M2 polarization, was also observed. Silencing of miR-155 was also associated with an increase in IL-4-induced M2 macrophage makers and a decreased expression of M1 macrophage markers [52].

Bao et al. [51] reported the interesting finding that miR-155 may target the *RELA* gene encoding p65, a subunit of NF-κB, which regulates the transcription of NLRP3 inflammasomes [51,53]. Moreover, levels of IL-6 and IL-1β secreted from cells transfected with miR-155 were decreased, which may indicate NF-κB signaling repression [51]. According to other studies, the expression of miR-155 is supposedly enhanced by Toll-like receptor activation of macrophages and dendritic cells, whereas the initiation of the immune reaction via the NF-κB pathway supposedly causes an enhanced expression of miR-155 [54]. It has been shown that in lymphocytes, miR-155 promotes Th1-dependent-responses, enhances proinflammatory cytokine secretion in myeloid cells and increases their proliferation [14]. Moreover, miR-155 deficiency among dendritic cells decreases their ability to activate T cells. MiR-155 inhibition was associated with decreased numbers of macrophages infiltrating the myocardium, as well as inhibited T cell activation accompanied by a decrease in cytokines such as TNF-α, IL-6, IL-10 and IFN-γ, which is consistent with findings from other studies [14,52]. It has been suggested that miR-155 may have a role in the regulation of B cell and T cell activation along with IL-10 [55,56]. Moreover, the inhibition of miR-155 resulted in Th17-related immune response suppression and decreased cardiac damage and decreased mortality due to VMC [14,56]. This phenomenon can be explained by the ability of miR-155 to decrease the expression of FoxP3 and ROR-γ-t within regulatory T cells (Treg). FoxP3 is responsible for the inhibition of the immune response as it regulates the differentiation of Tregs, whereas ROR-γ-t is a transcription factor responsible for Th17 lymphocyte differentiation. It is possible that an increased expression of miR-155 leads to a downregulation of FoxP3 within Tregs, which inhibits the Treg immune response and simultaneously promotes a Th17-mediated response [57]. An increased expression of miR-155 in myocarditis has also been observed within NK cells, which is correlated with an increased expression of IFN-γ [58]. Although miR-155 is claimed to have proinflammatory effects, which correlates with myocardial damage, it should be noted that increased inflammation may support pathogen clearance and alleviate myocarditis [58].

Data from the studies presented above suggest a proinflammatory role of miR-155, as it is involved in CD4+ and CD8+ T cell activation, the proliferation of splenic CD4+ T cells and the expression of proinflammatory IFN-γ, as well as in the induction of a proinflammatory pattern of macrophage proliferation. The expression of miR-155 is increased by Toll-like receptor activation. Moreover, miR-155 is involved in T cell and B cell activation. On the one hand, miR-155 may have a harmful effect on the myocardium in VMC, as it enhances inflammation; however, it is possible that enhanced inflammation supports pathogen clearance.

### 3.7. miR-146

Recent studies indicate that miR-146 has an ability to regulate immune responses in myocarditis. Increased levels of miR-146a within cardiomyocytes, as well as in serum, have been shown in CVB3-induced myocarditis [59,60]. Potential targets of miR-146a are TLR3, TRAF6 and IRAK1/2/4; however, TLR3 and TRAF6 have recently been shown to be relevant to myocarditis. TLR3 localized in the membrane of the endosome is activated by intracellular pathogen dsRNA. Its activation subsequently triggers NF-κB pathway activation via p65 and TRAF6. MiR-146a is suspected to target both p65 and TRAF6, leading to the suppression of NF-κB, which regulates the expression of proinflammatory IL-6 and TNF-**α** [59]. A potentially protective role of miR-146a in inflammation was also shown in a study by Feng et al. [61]. In a model of diabetic mice, the authors found that increased miR-146a protected the heart from inflammation, scarring and functional dysfunction. Reduced miR-146a levels were associated with an enhanced activation of the NF-κB pathway via IRAK1 and TRAF6, which were upregulated [61].

Similarly to miR-21, the expression of miR-146b has been found to be increased in VMC, whereas its silencing may decrease inflammation and hinder Th17 lymphocyte differentiation. The expression of miR-146b correlated with the previously described ROR-γ-t expression (see Section 3.2.). Moreover, a study by Obradovic et al. [38] showed only numerically higher levels among patients with inflamed cardiomyopathy compared to DCM patients; however, this observation did not reach statistical significance. Comparable results were obtained by Besler et al. [29], who revealed that endomyocardial miR-146b expression does not vary between inflammatory cardiomyopathy and DCM groups. Contrary results have been obtained in the pediatric population, revealing increased levels of miR-146b in the serum of patients with myocarditis [62,63].

Recently published studies have suggested different roles of miR-146a and miR-146b. Both subtypes tend to be increased in VMC; however, miR-146a indirectly decreases NF-κB pathway activation, which leads to anti-inflammatory effect, whereas miR-146b expression correlates with ROR-γ-t expression, leading to an increased differentiation of Th17 lymphocytes, which can be associated with enhanced Th17-mediated inflammation.

### 3.8. miR-203

Hemida et al. [64] have shown that CVB3 infection of the heart results in an increased activity of PKC and JunB, which is a positive transcription regulator of miR-203, whereas the levels of c-Jun, a repressor of miR-203 transcription, were decreased. The findings of their study indicate that zinc finger protein 148 (ZFP-148) is a target for miR-203. ZFP-148 is a transcription factor for cell cycle-regulating factors, including pro-apoptotic factors. Inhibition of ZFP-148 by miR-203 was associated with increases in cyclin D1, cyclin E, Bcl-2 and Bcl-Xl, whereas p21, p27, p53, Rb1 and Bcl-Xs remained decreased. These changes led to enhanced cell survival, which favors the replication of the virus [64].

In lipopolysaccharide-induced myocarditis, the expression of miR-203 tends to be increased, and miR-203 seems to promote apoptosis. Interestingly, it has been suggested that a decrease in miR-203 expression is associated with enhanced cell survival, decreases in caspase 3 and 3/7 activity, decreased cleaved-caspase 3 and a decline in cell apoptosis [65]. On the other hand, the overexpression of miR-203 prevents cardiomyocytes from undergoing apoptosis, which is consistent with the findings of Hemida et al. [64,65]. The protective role of miR-203 is weakened by the ability of miR-203 to target nuclear factor interleukin-3 (NFIL3), which plays an important role in cardiomyocyte survival regulation. An increased expression of NFIL3 results in the blockage of apoptosis and increased cardiomyocyte survival [65].

Currently available data suggest that the effect of miR-203 on cardiomyocytes in VMC depends on the model of VMC. Both models indicate that miR-203 tends to be increased in VMC regardless of the model (CVB3-induced vs. polysaccharide-induced). It is possible that miR-203 may increase cell survival, as it changes the levels of pro- and anti-apoptotic factors; however, due to its ability to target NFIL3, survival may be decreased. It is possible that cell survival depends on the balance between different pro- and anti-apoptotic factors.

### 3.9. miR-208

Circulating miR-208a has been found to be significantly increased in acute myocarditis caused by PVB19s, enteroviruses and adenoviruses. Upregulation of miR-208a occurs in response to myocardial damage, whereby miR-208b is associated with improper myocardial growth, cardiac remodeling and increased inflammation due to the overexpression of major histocompatibility complex molecules [66]. Calais et al. [67] have shown that in mice, miR-208a is able to cause cardiac hypertrophy and arrhythmias. On the other hand, it has been shown that miR-208a is crucial for proper heart development and impulse conduction, as it is involved in the expression of Cx40 [67].

A study by Corsten et al. [68] has shown increased serum levels of miR-208b in acute VMC; however, its upregulation may also be present in non-inflammatory acute heart failure. There are several potential targets for miR-208b; however, unfortunately, the role of these factors in VMC remains unclear and requires further studies. Overexpression of miR-208b was associated with cardiac hypertrophy, and a reduced expression of *MYH6* with an increased expression of *MYH7* encoding myosin-α and myosin-β, respectively. This phenomenon may suggest a significant role of miR-208b in cardiac remodeling. Moreover, the inhibition of miR-208b was associated with better cardiac function among patients with titin-based DCM [69]. In pediatric patients suffering from VMC caused by PVB19s, enteroviruses and adenoviruses, miR-208b levels in the subacute phase correlated with left ventricular systolic function in the resolution phase [66]. Thus, it is possible that miR-208b may play an important role in cardiac regeneration after myocarditis.

Both miR-208a and miR-208b tend to be increased in VMC. Overexpression of miR-208a in the acute phase of VMC may be associated with an increased risk of arrhythmias, whereas chronic upregulation may lead to cardiac hypertrophy. Moreover, an increased expression of miR-208b may constitute the underlying cause of cardiac hypertrophy and remodeling, as associations between changes in the expression of myosin-α and myosin-β and miRNA have been revealed.

### 3.10. miR-221 and miR-222

A protective role of miR-221/-222 cluster levels has been shown in CVB3-induced myocarditis. A study by Corsten et al. [70] revealed increased levels of the miR-221/-222 cluster in VMC, whereas the inhibition of these miRNAs was associated with an enhanced replication of CVB3 and increased inflammation. Moreover, the overexpression of miR-221/-222 was associated with decreased CVB3 viral load and prolonged viremia. Apart from potential virus cycle regulation by miR-221/-222, the cluster may also regulate the immune response. Changes in the immune response due to systemic inhibition of miR-221/-222 included an increased influx of macrophages and T lymphocytes. To explain the influence of miR-221/-222 on viral replication, as well as immune system modulation, several targets should be considered. These targets include *BMF*, *BCL2L11*, *ETS1*, *ETS2*, *CXCL12*, *IRF2* and *TOX.* The inhibition of *TOX*, *CXCL12* and *IRF* suppresses inflammation, which is consistent with the effect of miR-221/-222 and may explain its complex mechanism of action in patients with myocarditis [70].

### 3.11. miR-223

A study by Gou et al. [71] showed that in the heart tissue of CVB3-infected mice, expression of miR-223 was significantly reduced. A potentially cardioprotective role of miR-223 in myocarditis was suggested, based on the observation that the overexpression of miR-223 was associated with decreased inflammation, decreased mortality of CVB3-infected mice and increased LV systolic function. Moreover, the increased levels of cardiomyocyte damage biomarkers, as well as increased IFN-γ and IL-6, due to CVB3 infection were significantly reduced after inducing miR-223 overexpression, and an increase in anti-inflammatory IL-10 was observed. Interestingly, miR-223 also regulated cardiac macrophage polarization; miR-223 overexpression suppressed proinflammatory M1 macrophages and promoted anti-inflammatory M2 macrophage subtypes. The mechanism involved in macrophage polarization involves direct targeting of *Pknox1.* It was shown that an increased expression of *Pknox1* reverses the polarization pattern induced by miR-223 overexpression [71]. Furthermore, miR-223 has an ability to activate the PI3K/AKT signaling pathway, which contributes to anti-inflammatory effects through the negative regulation of the TLR4 signaling pathway. The TLR4 signaling pathway is a positive regulator of the immune response [72]. Thus, negative regulation of TLR4 may alleviate the immune response.

An interesting interaction between lncRNA maternally expressed 3 (MEG3), which tends to be overexpressed in VMC, and miR-223 in VMC was shown in a study by Xue et al. [73]. Downregulation of MEG3 is capable of inducing miR-223 expression, which targets *TRAF6*, whereas downregulation of TRAF6 is associated with NF-κB pathway inactivation. The inactivation of the NF-κB pathway promotes M2 macrophages and suppresses M1 macrophages. Thus, MEG3 and miR-223 may be responsible for maintaining the balance between the activation and suppression of the inflammatory response [73].

### 3.12. miR-590

Germano et al. [74] found that extracellular vesicles released from CVB3-infected cells are infectious and contain multiple overexpressed miRNAs. Among the miRNAs that were present within these extracellular vesicles, the highest expression level was found for miR-590-5p. The potential role of miR-590-5p in VMC was explained by its ability to target Spry1, a pro-apoptotic protein. Downregulation of Spry1 led to an increased activity of Akt, which promoted cell survival. The authors suggested that the presented mechanism may play an integral role in the early stages of infection, where infection via extracellular vesicles may dominate. It should be noted that prolonged cell survival due to miR-590-5p delivered within the vesicle contributes to prolonged vesicle release, which favors further spread of infection [74]. Another subtype of miR-590, miR-590-3p, targets p50, a subunit of NF-κB within inflamed cardiomyocytes [75].

### 3.13. miR-19

MiR-19a and miR-19b were shown to be upregulated in CVB3-induced myocarditis and to target the viral RNA-dependent RNA polymerase 3D, which increased viral RNA stability [76,77]. The suppression of miR-19b decreased viral replication and was associated with decreased viral load. Interestingly, Lin et al. [77] have shown that miR-19a may be involved in cardiac arrhythmias due to its direct targeting of gap junction protein α1 (GJA1). GJA1 decrease may constitute a substrate for arrhythmia, as it facilitates ventricular rendering, leading to re-entry formation. Moreover, it has been suggested that miR-19b may cooperate with miR-1, as they both target GJA1 [23,77].

Interestingly, contrary findings were presented by Jiahui et al. [78], in whose study the expression of miR-19b-3p in the hearts of CVB3-infected mice was decreased. The protective role of miR-19b-3p was confirmed with its upregulation, and the overexpression of miR-19b-3p was associated with a decrease in the biomarkers reflecting cardiomyocyte damage, decreased cytokine levels, increased cardiac systolic function and prolonged survival. Moreover, these authors found that in VMC, miR-19b-3p promotes M2 macrophage polarization, which reduces myocardial inflammation. The underlying mechanism of this phenomenon has not been fully explored; however, prediction analyses indicate that macrophage polarization may be mediated by the ability of miR-19b-3p to target PKNOX1 [78].

Current evidence suggests that miR-19a and miR-19b are increased in CVB3-induced myocarditis; however, their mechanisms of action are different. Overexpression of MiR-19a is potentially associated with an increased risk of cardiac arrhythmias, as it targets GJA1. On the other hand, the mechanism of action of miR-19b remains unknown; however, the suppression of this miRNA may suppress CVB3 replication. Thus, miR-19b may constitute a candidate for targeted treatment of CVB3-induced myocarditis.

### 3.14. MiRNAs and VMC in Non-CVB3 Models—Parvovirus B19 Myocarditis

In Europe, PVB19 is the most prevalent virus revealed in endomyocardial biopsies from patients with acute myocarditis and dilated cardiomyopathy with a suspected viral etiology; however, available data on the role of miRNA in PVB19-induced myocarditis remain scarce [79]. Due to differences in the pathophysiology of PVB19 infection as compared to CVB3, the authors have decided to present the mechanisms influenced by miRNAs in PVB19 separately.

PVB19 is considered to be a vasculotropic virus that infects the cardiac endothelial cells of venules, small arteries and arterioles; however, it has been shown that in fetal infections, PVB19 may directly infect myocardial fibers [80,81]. PVB19 enters the target cell via globoside 4 (Gb4) receptor, Ku80 receptor and integrin alfa-5beta. The cell infection process is highly regulated by TNF-**α**, which enhances Gb4 expression and activation [81]. Recent miRNA profiling in PVB19-induced myocarditis conducted by Kuhl et al. [82] has shown different miRNA expression profiles of 29 miRNAs in latent and active PVB19 infection of the myocardium. Prediction analysis has shown that all of 29 dysregulated miRNAs code for genes of cardiac diseases and immune response pathways [82]. Moreover, it has been shown that miRNA dysregulation in PVB19-induced VMC affects the expression of the *RORC* gene, as well as the gene encoding TNF-α, which play an important role in the immune response in VMC [32,82]. The role of the *RORC* gene, which encodes ROR-γ and ROR-γ-t, both of which are involved in Th17 lymphocyte differentiation, has been described in the section above (see Section 3.2. There is a lack of studies assessing miR-146b and miR-21 expression in in vitro models of PVB19 myocarditis; however, it is possible that miRNAs may act as a modulator of the inflammatory response by regulating Th17 lymphocyte differentiation in PVB19 myocarditis.

## 4. MicroRNA and the Diagnosis of Viral Myocarditis

Currently, endomyocardial biopsy (EMB) is still recognized as a gold standard for myocarditis diagnosis, as it is characterized by high sensitivity and specificity; however, it is an invasive method with limited accessibility [83]. Cardiac magnetic resonance (CMR) has been named the “non-invasive gold standard” for myocarditis diagnosis, as its accessibility is higher, but it has significant limitations, especially in terms of distinguishing between recent and remote inflammatory processes [84]. Thus, novel diagnostic tools are required to improve diagnostic performance in VMC, wherein miRNAs have been shown to have potential properties. It should be noted that miRNAs can be detected not only within cells but also in blood serum during VMC, wherein RNA-binding proteins and extracellular vesicles increase the stability of miRNAs [85,86]. The following summary of study results presents the current knowledge about the possibilities of using miRNAs in VMC diagnosis.

Blanco-Domínguez et al. [87] identified mmu-miR-721 as a potential biomarker of viral and autoimmune acute myocarditis in a mouse model. The researchers measured (with the use of microarrays and quantitative polymerase chain reactions) the level of circulating mmu-miR-721 in mouse plasma 10 days after Coxsackie virus infection. Levels of mmu-miR-721 were significantly higher when compared to the controls both in mice with severe heart inflammation (*p* = 0.02) and with less severe inflammation (*p* = 0.048). Although mmu-miR-721 is not present in humans, its homologue miR-Chr8:96 has been identified. Moreover, its potential to distinguish between myocarditis and diseases such as myocardial infarction, myocardial infarction with non-obstructive coronary arteries (MINOCA) and autoimmune diseases has been shown. The analysis of plasma from patients in the cohort revealed a significant increase in the expression of miR-Chr8:96 in patients with myocarditis (confirmed with EMB) when compared to healthy, STEMI and NSTEMI patients (*p* < 0.001). The diagnostic performance of miR-Chr8:96 expression measured in plasma was visualized by receiver operating characteristic (ROC) curves. In comparison with the myocardial infarction and control groups, the areas under the ROC curves (AUROC) for patients with myocarditis were 0.927 and 0.988, respectively [87].

Another study conducted by Aleshcheva et al. [88] identified six serum miRNAs which were significantly downregulated in non-viral chronic inflammation. Such results can enable the exclusion of a viral etiology. However, the lack of downregulation of these miRNAs may possibly increase the likelihood of a viral etiology. In the study, samples were identified by RNA isolation, reverse transcription, preamplification and expression analysis. MiR-1, miR-23, miR-142-5p, miR-155, miR-193 and miR-195 were downregulated in the non-viral chronic inflammation group in patients with inflammatory dilated cardiomyopathy when compared to the viral inflammation group (*p* < 0.01). At the same time, the study showed a high diagnostic value of these molecules in detecting inflammatory dilated cardiomyopathy (AUROC: 0.80–0.91). The obtained results indicate the potential role of these miRNAs in the diagnosis of myocarditis and in determining its etiology [88].

Kuehl et al. [89] identified miR-135b, miR-155, miR-190, miR-422a, miR-489, miR-590, miR-601 and miR-1290 as indicators of CVB3 cardiomyopathy with virus persistence and progressive clinical deterioration. However, their study was based on the profiling of miRNA isolated from endomyocardial biopsy [89]. An increase in miR-155 was also demonstrated in right ventricular septal biopsies from patients with acute VMC [14].

Some research leads us to presume that miR-30a may be a prospective biomarker of the immune response during Coxsackie heart infection [10], because it is upregulated in response to CVB3 infection [90,91]. Fan et al. [92] focused on patients with acute VMC (caused by CVB3) and examined various miRNAs with diagnostic utility in exosomes from serum samples. The levels of miRNAs in the serum exosome were measured by quantitative real-time polymerase chain reaction. It turned out that in the course of this disease, the expression of five miRNAs (miR-30a, miR-181d and miR-125a (upregulated) and miR-155 and miR-21 (downregulated)) was significantly changed (*p* < 0.05). Additionally, they examined miRNA expression in CVB3-infected HeLa cells and obtained a significant increase in the levels of miR-30a and miR-181d (*p* < 0.01), which may suggest that these two miRNAs may have particular potential in the diagnosis of acute VMC [92]. Interestingly, it was the only study which identified a decreased expression of miR-155 in VMC [90].

The level of miR-208a in plasma may be an indicator of the acute, subacute and chronic/resolution phases in child VMC. There was a 3,8-fold decrease and 3,1-fold decrease in miR-208b level, respectively, in the subacute and chronic/resolution phase in comparison to acute phase (*p* < 0.02). Similar properties were presented by miR-21. Both decreased significantly in the acute phase (*p* < 0.02); however, miR-21 did not change during the subacute phase. The researchers also showed that mirR-208b level during the subacute phase correlated with systolic left ventricular function during the chronic/resolution phase (*p* = 0.016) [66].

The most valuable markers for the diagnosis of virus-induced cardiac inflammation seem to be miR-Chr8:96, miR-155, mi-R30a and miR-181d (Table 2). In a systematic review of miRNAs as possible biomarkers for liquid biopsy (analysis of non-solid biological tissue, primarily blood; minimally invasive) in myocarditis, a detailed analysis of various studies allowed for the determination of the usability of individual miRNAs. They were divided into five utility groups (very low, low, medium, high, very high). Among those previously mentioned in the context of VMC, miR-155 and miR-Chr8:96 were placed in the group with very high utility as liquid biopsy markers. MiR-30a was in the group with high utility and miR-181d was assigned to the low-utility group [90].

**Figure 5:**
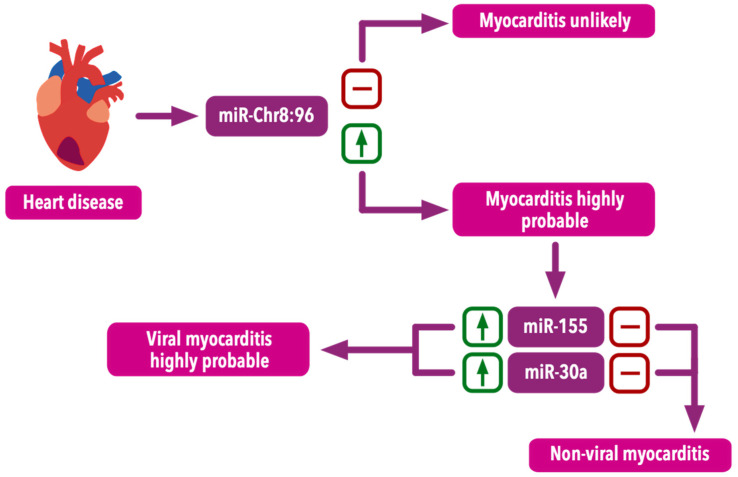
Potential diagnostic algorithm with the use micro-RNA measurements.

Recent data may be challenging to interpret due to the inconsistent results and distinct methodology of the cited studies. It should be noted that in some studies, miRNA was isolated from peripheral blood, and in others from biopsy tissue. The ability to obtain serum miRNAs whose levels are significantly associated with VMC diagnostic performance emphasizes the need for further studies to determine the exact role of miRNA in myocarditis diagnosis. For the implementation of the described methods, it is vital to define the guidelines for miRNA isolation, measurement methods, measurement units and other issues [85]. This analysis may help to outline the direction of further research (Figure 5).

## 5. Conclusions

Myocarditis constitutes a real diagnostic challenge for clinicians as there is a lack of specific and sensitive diagnostic tools and as its clinical manifestation can be highly heterogenous. Emerging data indicate an increasing role of ncRNAs, which are RNA subtypes that are not translated into proteins. To date, there has been a lack of studies that assess the role of ncRNAs in VMC; however, available data have revealed an important role of miRNAs, an ncRNA subtype, in VMC. It has been shown that miRNAs regulate the inflammatory reaction, apoptosis and necrosis and are involved in the regulation of cardioprotective mechanisms. It should be noted that numerous observations are based on animal studies; thus, the need for evaluation on human models should be emphasized. Moreover, the presented data open up a potential to assess several miRNAs in biopsies as well as in serum to support VMC diagnosis. Finally, increasing our understanding of the underlying mechanisms leading to VMC-induced cardiac damage may delineate future directions for drug development, as the activation or blockade of specific miRNAs may have an impact on disease course and prognosis.

## Figures and Tables

**Table 1:** Changes in miR-21 expression in viral myocarditis.

	Liu et al. [32]	He et al. [35]
miR-21 expression	Increased	Decreased in CVB3-induced VMC mice; the lowest expression was observed in severe VMC
Effect of miR-21 on VMC	miR-21 may aggravate VMC: Expression of miR-21 correlated with IL-17 and ROR-γ-t expression; Silencing of miR-21 reduced inflammatory lesions; inhibitors of miR-21 attenuated the severity of myocarditis	miR-21 may have a protective role in VMC: Expression of miR-21 was significantly lower in severe VMC when compared to benign VMC; miR-21 administration alleviated VMC
Time between infection and heart tissue collection	17 days	7 days
Animal model	BALB/c (H-2^d^) mice, 6 weeks of age	BALB/c (H-2^d^) mice, 6 weeks of age
Virus	CVB3 (Nancy strain), Hep-2 cell passage	CVB3 (Nancy strain), HeLa cell passage
RT-PCR primer sequence (5′-3′) for miR-21 expression analysis	Sense: TGACATCGCATGGCTGTA Antisense: GATGCTGGGTAATGTTTGAATG	Sense: GCGCTAGCTTATCAGACTGA Antisense: GTGCAGGGTCCGAGGT

CVB3—Coxsackie B3 virus; IL-17—interleukin 17; ROR—RAR-related orphan receptor; RT-PCR—real-time quantitative polymerase-chain reaction; miR-21—microRNA-21; VMC—viral myocarditis.

**Table 2:** Potential miRNA markers for the diagnosis of viral myocarditis.

miRNA Type	Utility (1–5)	Changes during Viral Infection	Paper
miR-Chr8:96	very high (5)	upregulated in viral and autoimmune acute myocarditis	Blanco-Domínguez et al. [87]
miR-155	very high (5)	upregulated in acute VMC	Corsten et al. [14]
		upregulated in viral cardiomyopathy	Kuehl et al. [89]
		downregulated in non-viral inflammatory dilated cardiomyopathy compared to inflammatory dilated cardiomyopathy with viral infection	Aleshcheva et al. [88]
		downregulated in acute VMC	Fan et al. [92]
miR-30a	high (4)	upregulated in acute VMC	Fan et al. [92]
		upregulated in response to CVB3 infection in vitro	Li et al. [91]
miR-181d	low (2)	upregulated in acute VMC	Fan et al. [92]

CVB—coxsackie B virus; microRNA—miR; VMC—viral myocarditis.

## Data Availability

The data used in this article were sourced from the materials mentioned in the References section.
